# Effects of 5-Hydroxymethylfurfural on In Vitro Rumen Fermentation Characteristics and Bacterial Community Structure of Diets with Different Concentrate-to-Roughage Ratios

**DOI:** 10.3390/ani16152446

**Published:** 2026-08-06

**Authors:** Xiaoxiao Du, Na Ren, Xianliu Wang, Jiaxin Qin, Wei Zhang, Liwen He

**Affiliations:** State Key Laboratory of Animal Nutrition and Feeding, College of Animal Science and Technology, China Agricultural University, Beijing 100193, China; 13664947616@163.com (X.D.); renna1231998@163.com (N.R.); wangxianliu@cau.edu.cn (X.W.); wzhang@cau.edu.cn (W.Z.)

**Keywords:** 5-HMF, concentrate-to-roughage ratio, in vitro rumen, bacterial community

## Abstract

To investigate the effects of 5-hydroxymethylfurfural (5-HMF) on ruminal fermentation characteristics and bacterial community structure, 5-HMF was supplemented at the levels of 0, 7500, and 30,000 mg/kg DM under two dietary concentrate-to-roughage ratios (20:80, T1; 80:20, T2) in in vitro rumen incubation, mainly focusing on gas production, fermentation parameters, nutrient digestibility and bacterial community. The results showed that 24 h gas production (GP_24_), 72 h gas production (GP_72_), the gas production rate (c) and dry matter digestibility (DMD) in T2 were higher than those in T1 (*p* < 0.01). With increasing levels of 5-HMF supplementation, GP_72_ and asymptotic gas production (B) increased linearly and quadratically (*p* < 0.05). T2 also had a higher butyrate concentration and a lower acetate/propionate (A/P) ratio than T1 (*p* < 0.05). With the increase in 5-HMF supplementation level, ruminal ammonia nitrogen (NH_3_-N) concentration showed a quadratic response. In contrast, the concentrations of total volatile fatty acids (TVFAs), acetate and isobutyrate decreased linearly. Propionate concentration showed linear and quadratic decreasing trends, while the A/P ratio exhibited linear and quadratic increasing trends (*p* < 0.05). The supplementation of 5-HMF at 30,000 mg/kg DM reduced propionate and isobutyrate concentrations under both dietary conditions (*p* < 0.05). No differences were observed in the alpha diversity indices of the bacterial community among different 5-HMF levels or substrates (*p* > 0.05). PCoA and PERMANOVA revealed that community structure differed between the 7500 mg/kg DM group and the 0 mg/kg DM group in the T1 diet (*p* = 0.011), whereas no difference was detected among T2 treatments. LEfSe revealed that 6 and 13 differential bacterial taxa were identified in T1 and T2 at the 5-HMF level of 7500 mg/kg DM, respectively. Correlation analysis revealed that, in T1, GP_72_ was positively correlated with *Pseudomonadota*, and the A/P ratio was positively correlated with fibrolytic taxa including *Rikenellaceae_RC9_gut_group* and *Christensenellaceae_R-7_group*. In T2, c was positively correlated with *Fibrobacterota* and *Fibrobacter*, while NH_3_-N was positively correlated with *Desulfovibrio*, indicating diet-specific microbial associations with rumen fermentation (*p* < 0.05). In conclusion, the high-concentrate diet exhibited stronger rumen fermentation activity. 5-HMF exerted diet-specific effects: In the low-concentrate diet, 5-HMF at 7500 mg/kg DM enriched fibrolytic microbes and shifted fermentation toward an acetate-type pattern. In the high-concentrate diet, it modulated hydrogen-metabolizing bacteria while selectively enriching *Pseudomonadota* capable of participating in carbohydrate metabolism. The 30,000 mg/kg DM level inhibited rumen fermentation. The findings provide a theoretical reference for the application of 5-HMF in ruminant feeds. However, further in vivo trials are required to validate its effects, and future studies with refined dose gradients based on realistic 5-HMF concentrations in feedstuffs are needed to comprehensively evaluate its efficacy and safety.

## Simple Summary

When feed ingredients are processed at high temperatures during manufacturing, a compound called 5-hydroxymethylfurfural (5-HMF) is produced and ends up in animal feed. While this compound is known to interfere with fermentation in other systems, very little is known about how it affects the rumen—the specialized stomach of cattle and sheep where microbes digest fibrous feed. In this study, we added different amounts of 5-HMF to two types of feed (one high in fiber and one high in grain) and incubated them with rumen fluid in the laboratory. We found that 7500 mg/kg DM 5-HMF modulated fiber digestion and influenced the abundance of certain bacterial taxa, while 30,000 mg/kg DM reduced overall fermentation activity. The effects also depended on the type of feed, with fiber-rich diets showing more pronounced responses. These findings suggest that 5-HMF is not simply harmful, and that its effects depend on both the amount present and diet composition. This knowledge can help the feed industry better understand how heat-processing by-products affect ruminant nutrition and guide the development of safer, more efficient feeding strategies.

## 1. Introduction

5-hydroxymethylfurfural (5-HMF) is a typical furan compound produced by acid-catalyzed dehydration of hexoses such as glucose and fructose under high temperature [1]. In biorefining, 5-HMF has long been recognized as a potent inhibitor of microbial fermentation. Multiple studies have demonstrated that 5-HMF markedly suppresses the activity of methanogens [2], reduces hydrogen production efficiency in dark fermentation systems [3], and inhibits ethanol fermentation by Saccharomyces cerevisiae [4]. Its inhibitory effect can be further intensified when combined with other fermentation by-products including levulinic acid and formic acid [5]. However, different microbial communities vary considerably in their tolerance and degradation capacity toward 5-HMF. For instance, anaerobic microbes can efficiently utilize 5-HMF in Pennisetum purpureum hydrolysate as a carbon source without compromising fermentation stability [6]. In contrast to its adverse effects in bioenergy systems, 5-HMF exhibits antioxidant, anti-inflammatory, and gut-modulatory properties in monogastric animals and mammals, improving growth performance and nutrient digestibility in weaned piglets and modulating intestinal microbial communities in carp [7,8]. These findings indicate that plant-derived natural products, including furan compounds, can regulate gastrointestinal microbiota, yet microbial responses to 5-HMF differ substantially across fermentation systems, limiting the generalizability of conclusions drawn from simple bioenergy fermentation models.

In the feed industry, thermal processing technologies such as steam explosion, extrusion, and high-temperature pelleting are widely employed to process unconventional feed ingredients, including low-quality crop straws and food by-products. These processes often generate substantial amounts of 5-HMF, which consequently becomes ubiquitous in animal feed [9,10]. For example, van Rooijen et al. [11] reported that canned pet foods contained an average of 1417 mg HMF per kg dry matter, while pelleted and extruded pet foods contained lower levels. In steam-exploded lignocellulosic biomass side streams, representing a more extreme thermal processing condition, 5-HMF concentrations can reach up to 9.0 g/kg dry matter [9]. The European Food Safety Authority (EFSA) has assessed the human health risks associated with furan compounds in foodstuffs and issued a scientific opinion regarding the animal health hazards of 5-HMF in bee feeds [12,13]. However, to date, no specific maximum acceptable levels for 5-HMF in ruminant feeds have been established. Moreover, given its demonstrated capacity to inhibit methanogenesis in other fermentation systems, the potential of 5-HMF to influence hydrogen metabolism in the rumen warrants further investigation [14]. Notably, 5-HMF is inherently a by-product formed during thermal processing, and it exerts dose-dependent biphasic biological effects: low concentrations may be metabolized by microorganisms or exert stimulatory impacts, while high concentrations can suppress microbial activity and even impair fermentation processes [2]. Given that rumen possesses a microbial ecosystem and fermentative physiology distinct from those of monogastric animals, systematic investigations into the dose–response effects of 5-HMF on rumen fermentation are of great significance for feed safety evaluation. Furthermore, the extensive adoption of high-concentrate finishing systems in modern ruminant production renders the dietary concentrate-to-roughage ratio a pivotal factor governing the composition and metabolic functions of ruminal microbiota [15]. Variations in dietary composition are likely to alter the susceptibility of ruminal microorganisms to 5-HMF, yet studies exploring the interactive effects between dietary structure and 5-HMF exposure have not yet been reported. Therefore, systematic exploration of the dose-dependent and diet-specific characteristics of 5-HMF in regulating rumen fermentation is essential for evaluating its feeding safety and application potential. Based on preliminary range-finding tests (5000 and 20,000 mg/kg DM), in which both tested levels increased gas production, two additional higher doses (7500 and 30,000 mg/kg DM) were adopted to further explore whether a more pronounced dose-dependent response would emerge. It was hypothesized that 5-HMF at 7500 mg/kg DM would modulate rumen fermentation and maintain microbial homeostasis, whereas at 30,000 mg/kg DM, it would exert inhibitory effects. Simultaneously, the dietary concentrate-to-roughage ratio would mediate these biological responses, leading to differentiated changes in rumen fermentation parameters and bacterial community profiles.

To verify these hypotheses, an in vitro rumen fermentation experiment was conducted to investigate the effects of 5-HMF on ruminal fermentation parameters and bacterial community structure under different dietary conditions and to clarify the interaction between dietary composition and 5-HMF supplementation level. This study aims to fill the research gap of 5-HMF in ruminant nutrition and provide a theoretical basis and experimental evidence for developing novel functional feed additives for ruminants.

## 2. Materials and Methods

### 2.1. Ethics Committee Approval

All the procedures with animals were approved by the China Agricultural University Laboratory Animal Welfare and Animal Experimental Ethical Inspection Committee (NO. AW82305202-2-5).

### 2.2. Experimental Materials and Design

The 5-HMF used in this study was purchased from Shanghai Macklin Biochemical Technology Co., Ltd. (Shanghai, China), with a purity of 95%. To determine the appropriate 5-HMF supplementation levels for the formal experiment, preliminary range-finding tests were first conducted. In these tests, 0.5% (5000 mg/kg DM) and 2% (20,000 mg/kg DM) 5-HMF were evaluated for their effects on gas production. The results showed that both doses increased gas production compared with the control. To further explore whether higher doses would induce different effects, two additional doses (7500 and 30,000 mg/kg DM) were selected for the formal experiment, representing a potentially modulatory level and a level expected to exert clear inhibition, respectively. Three supplemental levels of 5-HMF were applied to total mixed ration (TMR) substrates on a dry matter basis: 0, 7500 and 30,000 mg/kg DM, denoted as HMF0, HMF7500 and HMF30000, respectively. The experimental diets consisted of *Leymus chinensis* and commercial concentrate. Diet T1 comprised 80% *Leymus chinensis* (Gongzhuling Lvfeng Agriculture and Animal Husbandry Technology Development Co., Ltd., Jilin, China) and 20% commercial concentrate (Gongzhuling Hefeng Ruminant Feed Co., Ltd., Jilin, China). Diet T2 comprised 20% *Leymus chinensis* and 80% commercial concentrate. All feed samples were dried in a forced-air oven at 65 °C for 48 h to a constant weight, ground to pass through a 1 mm screen using a mill, and stored in sealed plastic bags for subsequent in vitro incubation. The nutrient composition of the two experimental substrates is presented in Table 1.

The in vitro culture was performed according to the procedure described by Menke et al. [16]. Three permanently rumen-fistulated Angus steers with an average body weight of 700 ± 25.84 kg and approximately 27 months of age were selected as rumen fluid donors. The steers were obtained from the China Agricultural University Beef Cattle Experimental Station (Doudian Hengsheng Animal Husbandry Technology Co., Ltd., Beijing, China). During the collection period, the steers were fed twice daily (at 08:00 and 16:00) with a diet consisting of a concentrate-to-roughage ratio of 56:44 (dry matter basis) and had free access to water. The detailed nutrient composition of the donor diet is presented in Table 2.

Rumen fluid was collected from each steer before the morning feeding. The collected rumen fluid was filtered through four layers of gauze, placed into a thermos flask, and immediately transported to the laboratory of China Agricultural University. Artificial rumen fluid was prepared by mixing fresh rumen fluid with buffer solution at a ratio of 1:2 (*v*/*v*) while being maintained in a 39 °C water bath and continuously aerated with CO_2_ throughout the preparation period. A 2 × 3 factorial design was adopted in this study, comprising two dietary treatments (T1 and T2) and three 5-HMF supplementation levels (0, 7500, and 30,000 mg/kg DM). All treatments were prepared from the same batch of substrates and incubated with the same batch of rumen fluid. For each treatment combination, six replicates were prepared, along with six blank controls (artificial rumen fluid without substrate) to correct for background interference. Based on this uniform experimental design, two separate assay systems were conducted in parallel to accommodate different incubation requirements and analytical endpoints: one for the measurement of gas production, fermentation parameters, and bacterial community composition using 100 mL glass syringes incubated for 72 h, and the other for the determination of dry matter digestibility (DMD) using incubation bottles incubated for 48 h. Both assay systems shared the identical treatment structure and blank controls. The two incubation durations were selected for their respective endpoints: 72 h for gas production to capture the complete fermentation profile until plateau, particularly for the high-roughage diet (T1), and 48 h for DMD determination, as this duration is widely adopted in standard in vitro digestibility protocols and has been shown to adequately capture differences among feedstuffs and treatments while providing a reliable estimate of in vivo digestibility [17,18].

For the gas production and fermentation parameters assay, 200 mg (dry matter basis) of each substrate was accurately weighed and placed into 100 mL glass syringes preheated to 39 °C. The 5-HMF was dissolved in distilled water to prepare stock solutions at 1.5 and 6.0 mg/mL, corresponding to final supplementation levels of 7500 and 30,000 mg/kg DM when 1 mL was added to 200 mg of substrate (dry matter basis). For the 0 mg/kg control, 1 mL of distilled water without 5-HMF was added. Each syringe then received 1 mL of the respective solution, followed by approximately 30 mL of artificial rumen fluid. In addition, six blank syringes containing only artificial rumen fluid without any substrate or 5-HMF were prepared to correct for gas production arising from the rumen fluid itself. All syringes were immediately sealed with rubber stoppers and incubated in a dark water bath at 39 °C for 72 h. Gas production was recorded at predetermined intervals throughout incubation, and the net gas production from each substrate was calculated by subtracting the gas production of the blank syringes. After 72 h, fermentation parameters and bacterial community composition were determined. For the DMD assay, 500 mg (dry matter basis) of each substrate was accurately weighed into incubation bottles pre-warmed to 39 °C. The corresponding 5-HMF solutions were added together with approximately 75 mL of artificial rumen fluid. Six blank bottles containing only artificial rumen fluid without any substrate or 5-HMF were also prepared. The bottles were sealed and incubated at 39 °C for 48 h, after which DMD was determined as described in Section 2.3.

### 2.3. Sample Collection and Analytical Procedures

Gas production was manually recorded at 0, 2, 4, 6, 8, 12, 16, 20, 24, 32, 40, 48, 60, and 72 h of incubation. After 72 h of incubation, fermentation was immediately terminated by placing the syringes in an ice-water bath. The final pH was measured using a portable pH meter (PHS-3C, Lei Magnetic Instrument Co., Ltd., Shanghai, China). The fermentation fluid was aliquoted into three 2 mL centrifuge tubes and stored at −20 °C until analysis. One aliquot was used for bacterial diversity analysis, and the other two were used for the determination of volatile fatty acids (VFA) and ammonia nitrogen (NH_3_-N). The concentration of NH_3_-N was determined using the colorimetric method described by Broderick and Kang [19]. The VFA concentration was analyzed using high-performance liquid chromatography (HPLC, Agilent 1260 Infinity II; Agilent Technologies, Waldbronn, Germany) [20]. DMD was determined as follows. After 48 h of incubation, the entire contents of each bottle were quantitatively transferred into pre-weighed nylon bags. The bags were rinsed with distilled water until the effluent was clear, dried at 65 °C for 48 h, and re-equilibrated in a desiccator for 24 h before weighing. The DMD was calculated as follows:

DMD (%) = (DM_1_ − DM_2_)/DM_1_ × 100 (1)

where DM_1_ is the dry matter weight of the substrate before fermentation (g), and DM_2_ is the dry matter weight of the residue remaining in the nylon bag after fermentation (g), calculated as the weight of the dried residue bag minus the weight of the empty bag. Blank bottles containing only artificial rumen fluid without substrate were included as controls, and the residual dry matter from the blanks was subtracted from the corresponding treatment groups to correct for the contribution of microbial precipitates and particulate matter from the rumen fluid.

### 2.4. Rumen Microbiota Analysis

During the 72 h incubation for gas production and fermentation parameter determination, three syringes were found to have leaked fermentation fluid, compromising sample integrity. These three samples (two from the T1 control group and one from the T1_HMF7500 group) were therefore excluded from subsequent DNA extraction and sequencing. Consequently, a total of 33 samples (rather than the planned 36) were subjected to 16S rRNA gene amplicon sequencing, with group sizes ranging from 4 to 6 replicates. To account for the unequal sample sizes, PERMANOVA (performed with 999 permutations in QIIME2) and two-way ANOVA (conducted using the GLM procedure in SAS) were applied, as both methods have been shown to be robust to moderate imbalances in group sample sizes.

Genomic DNA was extracted using a magnetic bead-based kit (DP712, Tiangen Biotech Co., Ltd., Beijing, China). The concentration of extracted DNA was quantified using a Qubit Fluorometer (Invitrogen, Carlsbad, CA, USA). The V3–V4 hypervariable region of the 16S rRNA gene was amplified using the universal primers 341F (5′ CCTACGGGNGGCWGCAG 3′) and 805R (5′ GACTACHVGGGTATCTAATCC 3′). The PCR products were purified using AMPure XP beads (Beckman Coulter, Brea, CA, USA), accurately quantified with the Qubit Fluorometer, and qualified using an Illumina library quantification kit (Kapa Biosciences, Wilmington, MA, USA). High-throughput sequencing was performed by Hangzhou Lianchuan Biotechnology Co., Ltd. (Hangzhou, China) on an Illumina NovaSeq 6000 platform using 2 × 250 bp paired-end sequencing.

### 2.5. Statistical Analysis

The in vitro gas production curves were fitted using the NLIN procedure of SAS 9.4 (SAS Institute Inc., Cary, NC, USA) according to the following model:

GP = B (1 − e^−ct^) (2)

where GP (mL/0.2 g DM) is the cumulative gas production at incubation time t; B (mL/0.2 g DM) is the asymptotic gas production; c (h^−1^) is the gas production rate; and t (h) is the in vitro incubation time.

Experimental data were statistically analyzed by analysis of variance (ANOVA) using the general linear model (GLM) procedure in SAS 9.4. The model was as follows:

Y_ij_ = μ + T_i_ + N_j_ + (T × N)_ij_ + e_ij_ (3)

where Y_ij_ is the dependent variable; μ is the overall mean; T_i_ is the fixed effect of different fermentation substrates (i = 1, 2); N_j_ is the fixed effect of 5-HMF treatment (j = 1, 2, 3); (T × N)_ij_ is the interaction effect between substrate and 5-HMF treatment; and e_ij_ is the random residual error.

Multiple comparisons were performed using Tukey’s test in SAS, and least-squares means (lsmeans) were used to calculate the standard error of the mean (SEM). Linear and quadratic effects were analyzed using the Contrast statement. Data are presented as means with SEM, and *p* < 0.05 was considered statistically significant. Bioinformatic analysis was conducted using the OmicStudio tools (https://www.omicstudio.cn/tool, accessed on 2 July 2026). Alpha diversity was calculated and displayed by QIIME2 (v2025.7, https://qiime2.org, accessed on 2 July 2026) and R software (version 4.3.1, R Foundation for Statistical Computing, Vienna, Austria), respectively. For beta diversity analysis, the Bray–Curtis distance matrix was calculated to reveal differences in microbial community structures across samples, followed by principal coordinate analysis (PCoA) for visualization. Permutational multivariate analysis of variance (PERMANOVA) implemented in QIIME2 was performed to test the statistical significance of intergroup discrepancies in community composition. A two-way ANOVA was adopted to compare the relative abundances of bacterial taxa across diets and 5-HMF supplementation levels. Linear discriminant analysis effect size (LEfSe) analysis was performed with an LDA score threshold of 3.0 to identify differentially enriched taxa [21]. Spearman correlation analysis was used to evaluate the relationships between ruminal microbiota and ruminal fermentation parameters. Spearman correlation analysis was performed using OriginPro 2024 (OriginLab Corporation, Northampton, MA, USA) to evaluate the relationships between ruminal bacterial taxa (at the phylum and genus levels) and in vitro gas production and fermentation parameters. Correlation coefficients (r) ranging from −1 to 1 were computed, with positive and negative correlations visualized in red and blue, respectively.

## 3. Results

### 3.1. In Vitro Ruminal Gas Production, Kinetic Parameters, and Dry Matter Digestibility

**Figure 1:**
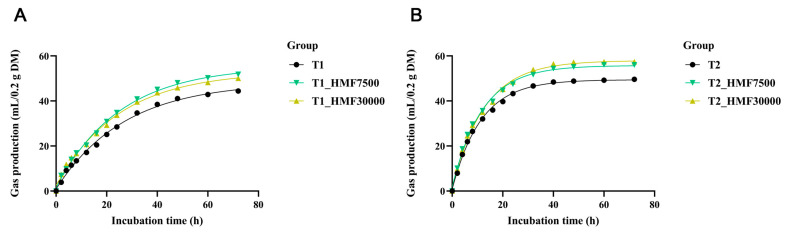
Cumulative gas production curves of substrates supplemented with different levels of 5-HMF. (**A**) Substrate T1. (**B**) Substrate T2. T1, T1_HMF7500, T1_HMF30000 and T2, T2_HMF7500, T2_HMF30000 represent the groups of two basal feed substrates (T1 and T2), with the 5-HMF contents in substrates set at 0, 7500, and 30,000 mg/kg DM, respectively.

As shown in Figure 1 (T1: A; T2: B) and Table 3, the GP_24_, GP_72_, c, and DMD were higher in T2 than in T1 (*p* < 0.01). The addition of 5-HMF increased GP_24_ for both substrates, with an interaction effect between substrate type and 5-HMF addition (*p* < 0.05). With increasing 5-HMF doses, GP_72_ and B increased both linearly and quadratically in both substrates (*p* < 0.05). Numerically, the highest values were observed at 7500 mg/kg DM for T1 and at 30,000 mg/kg DM for T2, although pairwise comparisons within each substrate revealed no differences among 5-HMF treatment groups in T1, and no difference between the 7500 and 30,000 mg/kg DM treatments in T2 (*p* > 0.05).

### 3.2. In Vitro Fermentation Parameters

The effects of different 5-HMF levels on in vitro fermentation parameters (pH, NH_3_-N, and VFA) at 72 h are summarized in Table 4. Neither substrate type nor 5-HMF supplementation affected the final pH (*p* > 0.05). The concentration of butyrate was higher in T2 than in T1, while the acetate-to-propionate (A/P) ratio was lower in T2 than in T1 (*p* < 0.05). Interactions between substrate type and 5-HMF addition were observed for NH_3_-N concentration and A/P ratio (*p* < 0.05). Quadratic effects of 5-HMF addition level were detected for NH_3_-N concentration, while both linear and quadratic effects were observed for the A/P ratio (*p* < 0.05). Specifically, in T1, the NH_3_-N concentration in the HMF7500 group (27.51 mg/dL) was higher than in the HMF0 and HMF30000 groups (both 23.98 mg/dL; *p* < 0.05). Supplementation with 5-HMF increased the A/P ratio in both substrates. In T1, the A/P ratio of the HMF7500 group was higher than that of the control group (*p* < 0.05), whereas no difference was observed between the HMF7500 and HMF30000 groups (*p* > 0.05). In T2, the HMF30000 group exhibited the highest A/P ratio, which was greater than that of both the HMF0 and HMF7500 groups (*p* < 0.05). In addition, with increasing 5-HMF supplementation levels, total volatile fatty acids (TVFAa), acetate, and isobutyrate concentrations decreased linearly, while propionate concentration decreased both linearly and quadratically (*p* < 0.05).

### 3.3. Bacterial Diversity

A total of 2,753,065 amplicon sequences were generated by amplicon sequencing in this study (83,426 ± 4030; *n* = 33). After quality control procedures including primer removal, denoising, sequence merging, and chimeric filtering, a total of 2,428,719 high-quality amplicon sequences were obtained (73,598 ± 3994; n = 33), with an average length of 410 base pairs. The Good’s coverage of each sample was greater than 0.99.

**Figure 2:**
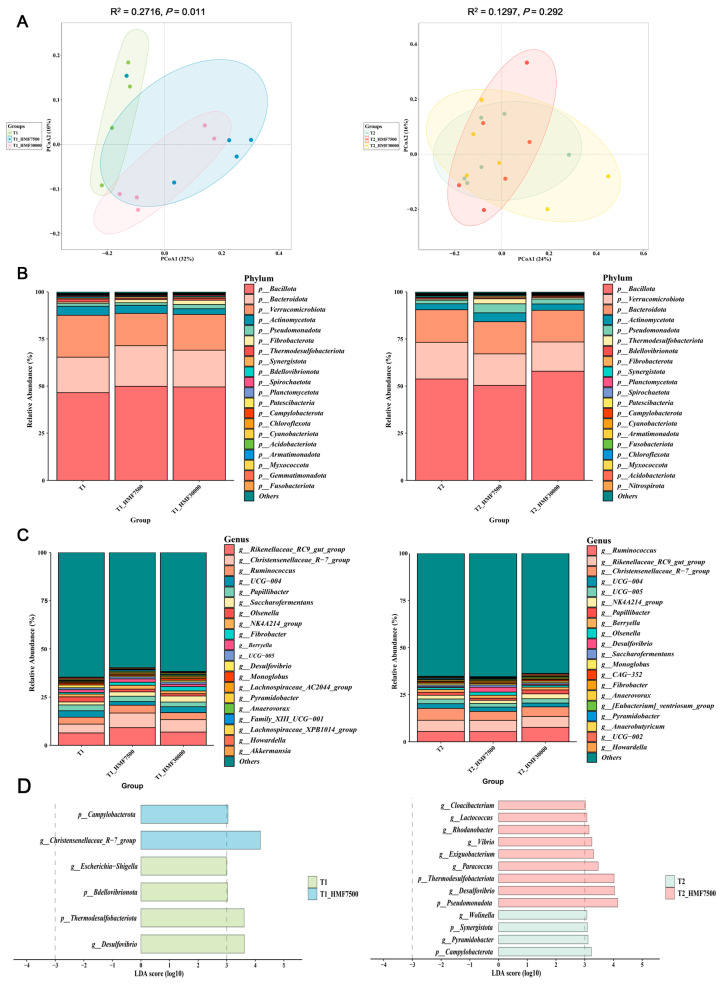
The effects of different feed substrates and 5-HMF levels on ruminal bacterial community structure. (**A**) Principal coordinate analysis (PCoA) based on the Bray–Curtis distance showing the beta diversity clustering of bacterial communities across treatments. Ellipses represent 95% confidence intervals. (**B**) Relative abundance of the top 20 dominant bacterial phyla. (**C**) Relative abundance of the top 20 dominant bacterial genera. (**D**) Linear discriminant analysis effect size (LEfSe) analysis identifying differentially abundant taxa between control and 5-HMF-treated groups (LDA score > 3.0, *p* < 0.05). Groups: T1 = diet with a concentrate-to-roughage ratio of 20:80; T2 = diet with a concentrate-to-roughage ratio of 80:20. HMF0, HMF7500 and HMF30000 correspond to 5-HMF supplemental levels of 0, 7500 and 30,000 mg/kg DM, respectively. The “Others” category covers taxa with a relative abundance lower than 0.01% or detected in fewer than half of all samples. Color coding: The groups are distinguished by the following colors: T1, grass green (#91be3e); T1_HMF7500, blue (#0081b4); T1_HMF30000, pink (#e990ab); T2, light grayish-blue (#96cbb3); T2_HMF7500, red (#e5352b); and T2_HMF30000, yellow (#ffd616).

Changes in the alpha and beta diversity of the bacterial community in response to 5-HMF levels during in vitro ruminal incubation are presented in Table 5 and Figure 2A, respectively. No differences were observed in the observed_features, ace, pielou_e, shannon, simpson, and faith-pd indices between the two dietary substrates (T1 and T2) for evaluating species diversity and richness (*p* > 0.05). Moreover, 5-HMF supplementation had no effect on the alpha diversity of ruminal microbiota (*p* > 0.05). PCoA based on Bray–Curtis distance was performed to evaluate the similarity or dissimilarity of bacterial community composition among different groups. In the T1 group, the principal coordinates PCo1 and PCo2 explained 32% and 10% of the total variation, respectively. In the T2 group, PCo1 and PCo2 explained 24% and 16% of the total variation, respectively. PERMANOVA further revealed that the T1_HMF7500 group differed from the T1 group (*p* = 0.011), indicating that 5-HMF treatment altered the ruminal bacterial community structure in T1. In T2, no difference in community structure was detected among treatments (*p* > 0.05).

Bacterial composition in the in vitro ruminal cultures at the phylum and genus levels is shown in Figure 2B,C, and Table 6 and Table 7. Taxonomic annotation identified a total of 37 phyla, among which 7 dominant phyla (relative abundance > 1%) were observed: *Bacillota* (50.26%), *Bacteroidota* (18.12%), *Verrucomicrobiota* (17.87%), *Actinomycetota* (3.91%), *Pseudomonadota* (4.52%), *Thermodesulfobacteriota* (1.22%), and *Fibrobacterota* (1.02%). At the genus level, 24 bacterial taxa had an average relative abundance greater than 1%. The top three dominant genera were *Rikenellaceae_RC9_gut_group* (6.48%), *Christensenellaceae_R-7_group* (5.62%), and *Ruminococcus* (4.89%).

The relative abundance of microbiota at the phylum and genus levels differed among treatments. At the phylum level, interactions between substrate and 5-HMF were observed on the relative abundances of *Pseudomonadota* and *Thermodesulfobacteriota* (*p* < 0.05). The relative abundances of *Bacteroidota* and *Fibrobacterota* were higher, while the relative abundance of *Pseudomonadota* was markedly lower in the T1 group than those in the T2 group (*p* < 0.05). With increasing supplemental levels of 5-HMF, the relative abundances of *Pseudomonadota* and *Thermodesulfobacteriota* exhibited quadratic responses (*p* < 0.05). The relative abundances of *Pseudomonadota* and *Thermodesulfobacteriota* were the highest in the T2_HMF7500 group, being higher than those in the T2 and T2_HMF30000 groups (*p* < 0.05). In contrast, 5-HMF supplementation decreased the relative abundance of *Thermodesulfobacteriota* in the T1 group (*p* < 0.05). At the genus level, interactions between substrate and 5-HMF were detected for the relative abundances of *Christensenellaceae_R-7_group*, *Saccharofermentans*, and *Desulfovibrio* (*p* < 0.05). Substrate type also affected the relative abundance of genera. Specifically, the relative abundances of *Rikenellaceae_RC9_gut_group*, *Papillibacter* and *Saccharofermentans* were higher in the T1 group, whereas the abundances of *Ruminococcus* and *UCG-005* were obviously lower compared with the T2 group (*p* < 0.05). The relative abundance of *Rikenellaceae_RC9_gut_group* and *Desulfovibrio* exhibited quadratic responses, while that of *Christensenellaceae_R-7_group* showed a linear effect with increasing 5-HMF dosage (*p* < 0.05). Meanwhile, the relative abundances of *Rikenellaceae_RC9_gut_group* and *Christensenellaceae_R-7_group* were higher in the T1_HMF7500 group than in the T1 group, and the relative abundance of *Desulfovibrio* was higher in the T2_HMF7500 group than in the T2 group (*p* < 0.05).

LEfSe analysis (LDA score > 3, *p* < 0.05) revealed distinct biomarker taxa between the control and 5-HMF treatment groups under different dietary substrates. A total of six differential bacterial taxa at the phylum and genus levels were identified between T1 and T1_HMF7500 groups, while 13 differential taxa were detected between T2 and T2_HMF7500 groups (Figure 2D). In the T1 substrate, the control group was enriched with *Thermodesulfobacteriota*, *Desulfovibrio*, *Escherichia-Shigella*, and *Bdellovibrionota*, whereas the T1_HMF7500 group specifically enriched *Christensenellaceae_R-7_group* and *Campylobacterota*. For the T2 substrate, the control group predominantly enriched *Campylobacterota*, *Synergistota*, *Pyramidobacter*, and *Wolinella*. In contrast, the T2_HMF7500 group exhibited enrichment of nine bacterial taxa, including *Thermodesulfobacteriota*, *Desulfovibrio*, *Pseudomonadota*, *Cloacibacterium*, *Lactococcus*, *Rhodanobacter*, *Vibrio*, *Exiguobacterium*, and *Paracoccus*.

### 3.4. Correlations Between Bacterial Taxa and In Vitro Gas Production and Fermentation Parameters

**Figure 3:**
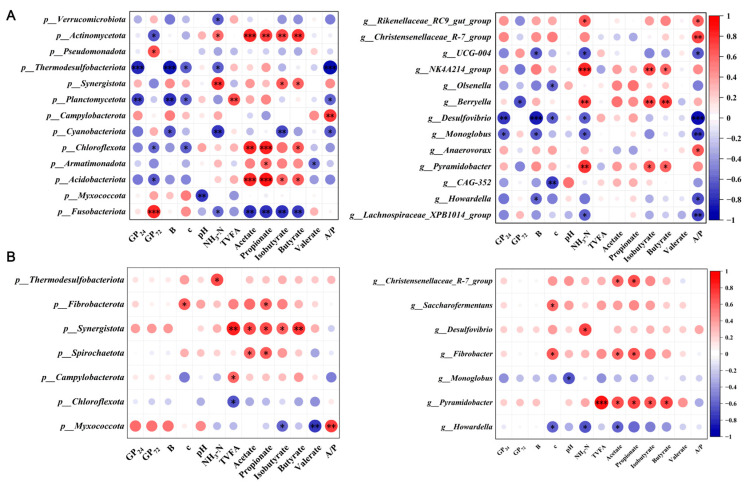
Correlation analysis between bacterial relative abundance at the phylum and genus levels and gas production and fermentation parameters within each diet. (**A**) T1 diet (concentrate-to-roughage ratio = 20:80); (**B**) T2 diet (concentrate-to-roughage ratio = 80:20). Spearman correlation coefficients (r) ranging from −1 to 1 were computed. Red and blue circles indicate positive and negative correlations, respectively. A single asterisk (*) in a circle indicates a weak correlation (*p* ≤ 0.05), a double asterisk (**) indicates a strong correlation (*p* ≤ 0.01), and a triple asterisk (***) indicates a very strong correlation (*p* ≤ 0.001). The size of each circle represents the magnitude of the correlation coefficient (|r|), with larger circles indicating stronger correlations. GP_24_: 24 h cumulative gas production; GP_72_: 72 h cumulative gas production; B: Asymptotic gas production of 0.2 g DM substrate; c: the rate of gas production per hour; NH_3_-N: ammonia nitrogen; TVFA: total volatile fatty acid; A/P: the ratio of acetate/propionate.

As shown in Figure 3, Spearman correlation analysis revealed significant correlations between bacterial taxa and in vitro ruminal gas production and fermentation parameters. For gas production indices, in the T1 diet, GP_24_ and B were negatively correlated with *Thermodesulfobacteriota*, *Planctomycetota*, *Desulfovibrio*, and *Monoglobus* (*p* < 0.05). GP_72_ was positively correlated with *Pseudomonadota* and *Fusobacteriota*, and negatively correlated with *Chloroflexota*, *Acidobacteriota*, and *Berryella* (*p* < 0.05). The gas production rate (c) was negatively correlated with *Thermodesulfobacteriota*, *Planctomycetota*, *Chloroflexota*, *Olsenella*, *Desulfovibrio*, and *CAG-352* in T1 (*p* < 0.05). In the T2 diet, c was positively correlated with *Fibrobacterota*, *Saccharofermentans*, and *Fibrobacter*, and negatively correlated with *Howardella* (*p* < 0.05).

For fermentation parameters, in the T1 diet, NH_3_-N concentration was positively correlated with *Actinomycetota*, *Synergistota*, *Rikenellaceae_RC9_gut_group*, *NK4A214_group*, *Berryella*, and *Pyramidobacter*, and negatively correlated with *Thermodesulfobacteriota*, *Cyanobacteriota*, *Fusobacteriota*, *UCG-004*, *Desulfovibrio*, *Monoglobus*, and *Lachnospiraceae_XPB1014_group* (*p* < 0.05). In the T2 diet, NH_3_-N concentration was positively correlated with *Thermodesulfobacteriota* and *Desulfovibrio*, and negatively correlated with *Howardella* (*p* < 0.05). In T1, acetate, propionate, isobutyrate, and butyrate were all positively correlated with *Actinomycetota* and *Acidobacteriota*, and negatively correlated with *Fusobacteriota* (*p* < 0.05). Additionally, acetate and propionate were positively correlated with *Chloroflexota*, while isobutyrate and butyrate were positively correlated with *Synergistota*, *NK4A214_group*, *Berryella*, and *Pyramidobacter* (*p* < 0.05). In T2, TVFA, acetate, propionate, isobutyrate, and butyrate were all positively correlated with *Synergistota* and *Pyramidobacter*; acetate and propionate were additionally positively correlated with *Spirochaetota*, *Christensenellaceae_R-7_group*, and *Fibrobacter* (*p* < 0.05). In T1, valerate was negatively correlated with *Armatimonadota*, whereas in T2, valerate was negatively correlated with *Myxococcota* (*p* < 0.05). The A/P ratio in T1 was positively correlated with *Campylobacterota*, *Rikenellaceae_RC9_gut_group*, *Christensenellaceae_R-7_group*, and *Anaerovorax*, and negatively correlated with *Thermodesulfobacteriota*, *Planctomycetota*, *Cyanobacteriota*, *UCG-004*, *Desulfovibrio*, *Monoglobus*, *Howardella*, and *Lachnospiraceae_XPB1014_group* (*p* < 0.05). In T2, the A/P ratio was positively correlated with *Myxococcota* (*p* < 0.05).

## 4. Discussion

### 4.1. Effects of 5-HMF on In Vitro Gas Production Kinetics and Dry Matter Digestibility of Diets with Different Concentrate-to-Roughage Ratios

Gas production and DMD are core indicators reflecting the extent of feed fermentation by rumen microorganisms, and are significantly negatively correlated with the fiber content of feed substrates [22]. In the present study, GP_24_, GP_72_, c, and DMD of the high-concentrate diet (T2) were significantly higher than those of the low-concentrate diet (T1), which is consistent with previous reports. The abundant fermentable carbohydrates such as starch in T2 can be rapidly utilized by rumen microorganisms, shortening the fermentation initiation time and improving substrate degradation efficiency [23]. Notably, the effect of 5-HMF supplementation on gas production showed a significant diet × dose interaction. GP_72_ and B peaked in the 7500 mg/kg DM group of T1, while the highest numerical values was observed in the 30,000 mg/kg DM group of T2, with both showing linear and quadratic increases. These results suggest that the response of rumen fermentation to 5-HMF differs depending on the dietary concentrate-to-roughage ratio. Ruminal gas production is closely associated with the degradation rate of dietary carbohydrates. The observed increases in gas production may be related to changes in the ruminal fibrolytic bacterial community in T1.

### 4.2. Effects of 5-HMF on In Vitro Fermentation Parameters of Diets with Different Concentrate-to-Roughage Ratios

Rumen pH, NH_3_-N, and VFA concentrations reflect rumen homeostasis and bacterial metabolic balance [24]. In the present study, 5-HMF supplementation had no significant effect on fermentation pH, and all treatments remained within the normal range of rumen pH (pH > 5.6) reported by Kleen et al. [25]. Dietary NH_3_-N concentration increased quadratically with increasing 5-HMF levels, and the NH_3_-N concentration in the 7500 mg/kg DM group of T1 was significantly higher than that in the control and 30,000 mg/kg DM groups. Rumen NH_3_-N concentration is jointly affected by ammonia release from protein degradation and ammonia utilization for bacterial crude protein (MCP) synthesis by rumen microorganisms. Given the increases in gas production, the elevated NH_3_-N concentration may be associated with increased degradation of crude protein in the roughage component of T1 [26,27]. Isobutyrate is a bacterial metabolite of valine in the rumen, accompanied by massive ammonia release, and their synchronous changes can serve as a specific indicator for evaluating in vitro protein degradation [28]. In the present study, isobutyrate and NH_3_-N concentrations in T1 reached the maximum at 7500 mg/kg DM 5-HMF, accompanied by significantly increased abundances of *Rikenellaceae_RC9_gut_group* and *Christensenellaceae_R-7_group*. This suggests that 5-HMF may promote bacterial deamination of protein in roughage, thereby improving ammonia release and utilization efficiency. As a high-concentrate diet, T2 provides sufficient nitrogen sources for bacterial growth, with active protein degradation and assimilation processes, resulting in a relatively stable NH_3_-N concentration without significant differences among treatments [29].

VFA are produced by bacterial fermentation of carbohydrates, and their profile reflects rumen fermentation type, accounting for more than 70% of the energy supply for ruminants. Fermentation is also accompanied by the release of gases such as H_2_, CO_2_, and CH_4_ [30]. In this study, VFA concentrations decreased to varying degrees after 5-HMF supplementation, which is inconsistent with the conventional understanding that increased gas production is usually accompanied by elevated VFA concentrations. This discrepancy may be explained by two factors. First, 5-HMF may be anaerobically reduced into less toxic furan alcohol intermediates by enteric microbes, and these intermediate products can be further mineralized to CH_4_ and CO_2_ [31,32]. Second, the increased gas production may reflect altered carbon or hydrogen metabolism rather than enhanced fermentation efficiency. Since gas composition was not measured, the origin of the additional gas remains unclear. Future studies measuring gas composition (CH_4_, H_2_, CO_2_) are needed to fully evaluate the metabolic consequences of 5-HMF supplementation.

In T1, 5-HMF supplementation linearly and quadratically increased the ratio of A/P accompanied by reduced propionate concentration, indicating that 5-HMF shifted ruminal carbohydrate fermentation toward an acetate-type pattern, providing a fermentation basis for increased gas production [18]. Bacterial community analysis showed that 5-HMF supplementation significantly enriched *Christensenellaceae_R-7_group* while reducing the hydrogen-utilizing bacterium *Desulfovibrio*. Ma et al. [33] reported that elevated rumen dissolved H_2_ in dairy cows led to succinate accumulation and increased A/P ratio, and the enrichment of *Christensenellaceae* was closely related to this process. The decreased propionate concentration may be associated with inhibited conversion of succinate to propionate. Based on these results, we speculate that 5-HMF may regulate hydrogen metabolism-related microbiota, redirecting metabolic hydrogen toward succinate and gas production pathways rather than propionate synthesis, thereby reducing propionate production, increasing A/P ratio, and enhancing gas production. However, since the concentrations of succinic acid and different gas components were not directly measured in this study, the above speculation remains to be further verified.

Although TVFA concentrations also declined in T2 after 5-HMF supplementation, individual VFA contents were higher in the 7500 mg/kg DM group than in the 30,000 mg/kg DM group, accompanied by a marked increase in the relative abundance of *Pseudomonadota*. The phylum *Pseudomonadota* is a large and functionally diverse group. Previous studies have identified *Pseudomonadota* as a dominant phylum in lignocellulolytic microbial consortia derived from the rumen, and metaproteomic analyses have revealed its involvement in the production of carbohydrate-active enzymes and biomass deconstruction [34]. In addition, Crigler et al. [35] demonstrated that *Pseudomonas putida ALS1267*, a member of this phylum, possesses a complete metabolic pathway for 5-HMF, enabling its utilization as a carbon source. Based on these observations, we speculate that the enrichment of *Pseudomonadota* observed in the present study may be beneficial for carbohydrate degradation in the rumen. However, whether certain members of this phylum are capable of transforming or utilizing 5-HMF remains to be determined. In addition, supplementation with 7500 mg/kg DM 5-HMF significantly increased the relative abundance of *Desulfovibrio* in T2. As one of the predominant sulfate-reducing bacteria in the rumen, *Desulfovibrio* utilizes hydrogen ions to reduce sulfate and competes with methanogens for hydrogen substrates, thereby directly mitigating methane production. Accordingly, promoting the proliferation of *Desulfovibrio* via high-sulfur diets has been proposed as a promising strategy for methane abatement [36,37]. In this experiment, 5-HMF enriched *Desulfovibrio* under the T2 diet. It is plausible that 5-HMF may have selectively modulated ruminal microbiota to alleviate nutritional competition for *Desulfovibrio* and facilitate its growth. However, since CH_4_ and H_2_S were not directly measured in this study, the potential role of 5-HMF in mitigating methane emissions remains speculative and warrants further investigation.

Overall, when the 5-HMF dosage reached 30,000 mg/kg DM, the concentrations of individual VFAs in ruminal fermentation liquor were lower than those in the control group and the 7500 mg/kg DM group across both diets. This phenomenon may be attributed to the inhibition of certain bacterial communities and related enzymatic activities, while the underlying biological mechanisms remain unclear. The significant interaction between 5-HMF and diet type further verifies that the regulatory effects of 5-HMF on VFAs depend on fermentation substrates. Hence, further studies with refined dose gradients are needed to identify the appropriate 5-HMF supplementation level according to the basal diet of ruminants.

### 4.3. Effects of 5-HMF on Ruminal Bacterial Communities of Diets with Different Concentrate-to-Roughage Ratios

The diversity and structural stability of the rumen bacterial community are fundamental for normal fermentation function. In this study, 5-HMF supplementation did not significantly affect bacterial alpha diversity, indicating that it did not disrupt the richness and evenness of the rumen bacterial community. This result is consistent with the unchanged pH. However, PCoA and PERMANOVA based on the Bray–Curtis distance showed that the HMF7500 group differed significantly from other groups in T1, whereas no significant difference was observed among treatments in T2. These results indicate that 5-HMF affects bacterial community structure under low-concentrate diets, whereas the abundant fermentable carbohydrates in high-concentrate diets may buffer the impact of 5-HMF on microbiota.

Taxonomic analysis showed that rumen bacteria were dominated by *Bacillota* and *Bacteroidota* at the phylum level, accounting for 58%~75% of the total relative abundance, which is consistent with previous findings [38]. *Bacillota* plays a key role in degrading cellulose and hemicellulose, while *Bacteroidota* is central to the degradation of dietary protein and non-structural carbohydrates [39,40]. At the genus level, the dominant taxa were *Rikenellaceae_RC9_gut_group*, *Christensenellaceae_R-7_group*, and *Ruminococcus*. The T1 group was significantly enriched with fibrolytic microorganisms including *Rikenellaceae_RC9_gut_group*, *Papillibacter* and *Saccharofermentans*. These microbes are characterized by efficient cellulose degradation and acetate production, enabling them to thrive well under high-fiber dietary conditions [41]. In contrast, *Ruminococcus* and *UCG-005*, which preferentially utilize non-structural carbohydrates such as starch, were predominant in the T2 group [42]. These findings indicate that dietary concentrate-to-roughage ratio selectively screens functional ruminal microbiota depending on substrate types, thereby shaping distinct bacterial community structures.

For T1, supplementation with 5-HMF at 7500 mg/kg DM significantly increased the relative abundances of *Rikenellaceae_RC9_gut_group* and *Christensenellaceae_R-7_group*. These two genera can efficiently degrade fibrous polysaccharides and proteins and produce acetate, so their elevated abundance reflected enhanced carbohydrate degradation capacity in the rumen [43]. Correlation analysis showed that the A/P ratio was significantly positively correlated with *Rikenellaceae_RC9_gut_group* and *Christensenellaceae_R-7_group*, which supported the highest acetate-to-propionate ratio observed in the T1_HMF7500 group. Likewise, the relative abundances of *Fibrobacterota*, *Fibrobacter* and *Saccharofermentans* tended to increase under the 30,000 mg/kg DM 5-HMF treatment. This confirmed that 5-HMF could modulate ruminal fiber degradation and the utilization efficiency of roughage [44,45].

In T2, 5-HMF supplementation at 7500 mg/kg DM significantly enriched *Desulfovibrio*. As a typical sulfate-reducing bacterium, *Desulfovibrio* has much higher thermodynamic and kinetic affinity for hydrogen than methanogens, and thus competes for hydrogen preferentially. The reduced partial pressure of hydrogen can further inhibit the hydrogen-dependent propionate synthesis pathway [46,47]. Acetate is mainly produced via direct hydrolytic fermentation of cellulose and is less susceptible to hydrogen partial pressure. Consequently, propionate content decreased to a greater extent than acetate, ultimately leading to an increased acetate-to-propionate ratio. High concentrations of hydrogen sulfide can damage the ruminal epithelial barrier and even induce sulfur toxicity in ruminants [36]. In the present study, stable fermentation status was observed in all groups without any adverse symptoms. Meanwhile, the relative abundance of *Pseudomonadota* increased obviously in this treatment group. This phylum has been proven to possess fibrolytic capacities [34]. This suggested that 5-HMF supplementation in T2 could contribute to substrate utilization. Consistently, high doses of 5-HMF significantly reduced total volatile fatty acid concentrations in both diets, implying that the overall rumen fermentation activity was suppressed.

In summary, the regulatory effects of 5-HMF on rumen microbiota and fermentation functions were strongly dependent on dietary composition. Overall, 5-HMF did not disrupt rumen bacterial diversity or internal homeostasis, but selectively altered bacterial community structure. In low-concentrate and high-fiber diets, 5-HMF mainly enriched fibrolytic and acetate-producing functional bacteria, which may facilitate crude fiber degradation and shift fermentation toward an acetate-type pattern. Within the dose range tested in this study, fibrolytic taxa consistently showed enrichment trends. In high-concentrate diets, 5-HMF primarily regulated hydrogen-metabolizing microbiota. Supplementation with 7500 mg/kg DM of 5-HMF enriched *Desulfovibrio*, which is hypothesized to inhibit propionate synthesis via hydrogen competition, potentially altering metabolic flux. Meanwhile, the enrichment of *Pseudomonadota* suggested modulation of substrate degradation. However, a dosage of 30,000 mg/kg DM suppressed overall rumen fermentation activity. It should be noted that the six replicates per treatment were derived from a single pooled rumen inoculum incubated on a single occasion; thus, they represent analytical replicates rather than independent experimental units, which should be considered when generalizing the statistical inferences.

## 5. Conclusions

This study demonstrates that the regulatory effects of 5-HMF on rumen fermentation and microbiota are dose-dependent and diet-specific. Within the scope of this study, 7500 mg/kg DM maintained rumen microecological homeostasis and modulated fermentation toward an acetate-type pattern, whereas 30,000 mg/kg DM was shown to suppress overall fermentative activity. Under a low-concentrate diet (concentrate-to-roughage ratio = 20:80), 5-HMF primarily enriches fiber-degrading bacteria, which may modulate fiber degradation. Under a high-concentrate diet (concentrate-to-roughage ratio = 80:20), the abundant starch-based substrates buffer the impact of 5-HMF on bacterial community structure; an appropriate 5-HMF level enriches *Desulfovibrio*, thereby altering ruminal hydrogen flow and volatile fatty acid composition, while also inducing the enrichment of *Pseudomonadota*, which can metabolize carbohydrates and enhance energy supply. Collectively, the biological effects of 5-HMF are co-modulated by the supplementation dose and the dietary concentrate-to-roughage ratio. Based on the experimental conditions and results of the present study, 7500 mg/kg DM was the level at which fiber-associated and 5-HMF-metabolizing taxa were most enriched under both dietary regimes. In practical application, the 5-HMF dosage should be matched with the specific diet composition. This study elucidates the effects of 5-HMF on in vitro fermentation characteristics and rumen microbiota under different concentrate-to-roughage ratios, providing a reference for the scientific application of 5-HMF in ruminant feed. Future in vivo feeding trials are warranted to further validate its long-term effects on animal performance.

## Figures and Tables

**Table 1:** Ingredients and proximate composition of fermentation substrate.

Items	T1	T2
Ingredients (% DM basis)		
Concentrate supplement ^1^	20	80
*Leymus chinensis*	80	20
Proximate composition (% DM basis)		
Dry matter	94.78	93.77
Ash	6.26	7.87
Ether extract	1.57	2.74
Crude protein	8.70	15.84
Neutral detergent fiber	58.18	32.88
Acid detergent fiber	39.50	17.52

^1^ Main ingredients of concentrate supplement: corn, soybean meal, cottonseed meal, peanut meal, wheat bran, Distillers Dried Grains with Solubles (DDGSs), corn germ meal, corn germ cake, sprayed corn hull, glucose, magnesium oxide, salt, sodium bicarbonate, and premix.

**Table 2:** Diet formulation and nutrient levels of donor diet for fistulated cattle.

Items	Content
Ingredients (% DM basis)	
Corn silage	31.00
Wheat straw	13.00
Ground corn	32.00
Jujube powder	6.00
Soybean meal	8.00
Palm meal	6.00
Premix ^1^	1.70
Sodium bicarbonate	0.80
Salt	0.50
Limestone	1.00
Nutrient level (% DM basis)	
Crude protein	11.61
Ether extract	3.15
Neutral detergent fiber	33.38
Acid detergent fiber	20.37
Calcium	0.59
Phosphorus	0.27

^1^ The premix provided the following per kg of diet: vitamin A acetate 150,000–450,000 IU; vitamin D_3_ 40,000–120,000 IU; Cu 250–750 mg; Fe 1000–5000 mg; Mn 1000–3000 mg; Zn 1500–3700 mg; Ca 10–25%; total *p* ≥ 0.3%; NaCl 15–30%; moisture ≤ 12%.

**Table 3:** Effect of 5-HMF addition on gas production kinetics parameters and dry matter digestibility of different substrates.

Items ^1^	Diet	Groups ^2^			SEM	*p* ^3^				
		HMF0	HMF7500	HMF30000		T	H	L	Q	T × H
GP_24_, mL/0.2 g DM	T1	28.46 ^Bb^	40.86 ^a^	33.68 ^Bb^	1.536	<0.001	<0.001	0.572	0.089	0.016
	T2	43.29 ^Ab^	47.49 ^ab^	48.48 ^Aa^						
GP_72_, mL/0.2 g DM	T1	44.44 ^B^	51.82	50.09 ^B^	1.135	0.005	0.004	0.044	0.049	0.753
	T2	49.65 ^Ab^	56.05 ^ab^	57.41 ^Aa^						
B, mL/0.2 g DM	T1	47.45	53.94	51.27	1.028	0.093	0.008	0.033	0.032	0.380
	T2	49.08 ^b^	55.18 ^ab^	57.57 ^a^						
c, h^−1^	T1	0.040 ^B^	0.043 ^B^	0.047 ^B^	0.005	<0.001	0.949	0.766	0.752	0.291
	T2	0.093 ^A^	0.093 ^A^	0.087 ^A^						
DMD, %	T1	56.60 ^B^	59.91 ^B^	59.34 ^B^	2.596	<0.001	0.142	0.423	0.606	0.180
	T2	84.05 ^A^	84.09 ^A^	84.26 ^A^						

^1^ Items, GP_24_: 24 h cumulative gas production; GP_72_: 72 h cumulative gas production; B: asymptotic gas production of 0.2 g DM substrate; c: the rate of gas production per hour; DMD: dry matter digestibility. ^2^ Groups HMF0, HMF7500, and HMF30000 indicate that the content of 5-HMF in the fermentation substrate is 0, 7500, and 30,000 mg/kg DM, respectively. ^3^ *p*, T: substrate; H: 5-HMF; L: linear; Q: quadratic; T × H: the interaction of substrate and 5-HMF. ^A–B^ In each column, mean values with different uppercase superscripts (A,B) indicate significant differences between dietary treatments (*p* < 0.05). ^a–b^ In each row, mean values with different lowercase superscripts (a–b) indicate significant differences among 5-HMF supplementation levels (*p* < 0.05).

**Table 4:** Effect of 5-HMF addition on fermentation characteristics of different substrates.

Items ^1^	Diet	Groups ^2^			SEM	*p* ^3^				
		HMF0	HMF7500	HMF30000		T	H	L	Q	T × H
pH	T1	7.11	7.09	7.09	0.018	0.820	0.829	0.551	0.752	0.779
	T2	7.07	7.07	7.12						
NH_3_-N, mg/dL	T1	23.98 ^b^	27.51 ^a^	23.98 ^b^	0.309	0.730	0.006	0.511	0.010	0.020
	T2	25.08	25.53	25.38						
TVFA, mmol/L	T1	83.95 ^a^	79.77 ^ab^	73.19 ^b^	1.440	0.748	0.004	<0.001	0.604	0.909
	T2	86.02	80.74	72.56						
Acetate, mmol/L	T1	56.91	55.50	50.76	1.077	0.202	0.011	0.004	0.410	0.711
	T2	56.51	51.65	48.35						
Propionate, mmol/L	T1	16.03 ^a^	14.15 ^b^	13.26 ^b^	0.363	0.352	<0.001	<0.001	0.031	0.566
	T2	17.04 ^a^	14.63 ^ab^	13.06 ^b^						
Isobutyrate, mmol/L	T1	1.03 ^ab^	1.12 ^a^	0.88 ^b^	0.039	0.637	<0.001	<0.001	0.096	0.147
	T2	1.20 ^a^	1.13 ^a^	0.77 ^b^						
Butyrate, mmol/L	T1	8.63 ^B^	8.67	8.10	0.178	0.005	0.141	0.179	0.435	0.965
	T2	9.58 ^A^	9.47	8.91						
Valerate, mmol/L	T1	1.35	1.27	0.74	0.117	0.162	0.918	0.496	0.122	0.761
	T2	1.69	1.99	1.96						
A/P	T1	3.55 ^Ab^	3.92 ^Aa^	3.83 ^Aa^	0.037	<0.001	<0.001	<0.001	0.041	<0.001
	T2	3.32 ^Bc^	3.53 ^Bb^	3.70 ^Ba^						

^1^ Items: NH_3_-N: ammonia nitrogen; TVFA: total volatile fatty acid; A/P: the ratio of acetate/propionate. ^2^ Groups HMF0, HMF7500, and HMF30000 indicate that the content of 5-HMF in the fermentation substrate is 0, 7500, and 30,000 mg/kg DM, respectively. ^3^ *p*, T: substrate; H: 5-HMF; L: linear; Q: quadratic; T × H: the interaction of substrate and 5-HMF. ^A–B^ In each column, mean values with different uppercase superscripts (A,B) indicate significant differences between dietary treatments (*p* < 0.05). ^a–c^ In each row, mean values with different lowercase superscripts (a–c) indicate significant differences among 5-HMF supplementation levels (*p* < 0.05).

**Table 5:** Effects of different feed substrates and 5-HMF levels on alpha diversity of rumen bacterial communities.

Items	Diet	Groups ^1^			SEM	*p* ^2^				
		HMF0	HMF7500	HMF30000		T	H	L	Q	T × H
observed_features	T1	1744	1680	1657	47.549	0.065	0.876	0.671	0.934	0.931
	T2	1507	1532	1471						
ace	T1	1848	1783	1756	54.106	0.068	0.871	0.677	0.933	0.957
	T2	1586	1611	1538						
pielou_e	T1	0.910	0.906	0.906	0.002	0.153	0.738	0.602	0.598	1.002
	T2	0.903	0.900	0.900						
shannon	T1	9.768	9.678	9.684	0.060	0.086	0.849	0.603	0.967	0.934
	T2	9.518	9.518	9.417						
simpson	T1	1.000	1.000	1.000	0.001	0.129	0.425	0.348	0.316	0.429
	T2	0.998	1.000	0.997						
faith-pd	T1	150.988	147.652	146.104	3.428	0.529	0.071	0.729	0.983	0.918
	T2	135.570	135.890	132.708						

^1^ Groups HMF0, HMF7500, and HMF30000 indicate that the content of 5-HMF in the fermentation substrate is 0, 7500, and 30,000 mg/kg DM, respectively. ^2^ *p*, T: substrate; H: 5-HMF; L: linear; Q: quadratic; T × H: the interaction of substrate and 5-HMF.

**Table 6:** Relative abundance of bacterial phyla in rumen cultures in vitro (relative abundance > 1%).

Items	Diet	Groups ^1^			SEM	*p* ^2^				
		HMF0	HMF7500	HMF30000		T	H	L	Q	T × H
*Bacillota*	T1	46.54	49.85	49.20	1.670	0.113	0.641	0.685	0.396	0.586
	T2	53.79	50.57	57.94						
*Bacteroidota*	T1	19.06	21.61 ^A^	19.55	0.724	0.043	0.732	0.472	0.912	0.754
	T2	17.16	17.07 ^B^	16.77						
*Verrucomicrobiota*	T1	22.29	17.28	19.17	1.384	0.430	0.501	0.380	0.416	0.909
	T2	19.55	16.63	15.50						
*Pseudomonadota*	T1	1.41	1.28 ^B^	2.26	0.272	0.001	0.015	0.487	0.028	0.002
	T2	1.85 ^b^	4.79 ^Aa^	2.71 ^b^						
*Actinomycetota*	T1	4.62	4.29	3.09	0.215	0.529	0.052	0.074	0.103	0.190
	T2	3.28	4.61	3.35						
*Thermodesulfobacteriota*	T1	1.55 ^a^	0.77 ^c^	1.07 ^b^	0.185	0.373	0.051	0.388	0.036	0.001
	T2	0.65 ^b^	2.72 ^a^	0.77 ^b^						
*Fibrobacterota*	T1	0.79	1.68	2.31 ^A^	0.188	0.003	0.140	0.285	0.201	0.149
	T2	0.44	0.85	0.43 ^B^						

^1^ Groups HMF0, HMF7500, and HMF30000 indicate that the content of 5-HMF in the fermentation substrate is 0, 7500, and 30,000 mg/kg DM, respectively. ^2^ *p*, T: substrate; H: 5-HMF; L: linear; Q: quadratic; T × H: the interaction of substrate and 5-HMF. ^A–B^ In each column, mean values with different uppercase superscripts (A,B) indicate significant differences between dietary treatments (*p* < 0.05). ^a–c^ In each row, mean values with different lowercase superscripts (a–c) indicate significant differences among 5-HMF supplementation levels (*p* < 0.05).

**Table 7:** Relative abundance of bacterial genera in rumen cultures in vitro (relative abundance > 1%).

Items	Diet	Groups ^1^			SEM	*p* ^2^				
		HMF0	HMF7500	HMF30000		T	H	L	Q	T × H
*Rikenellaceae_RC9_gut_group*	T1	6.57 ^b^	9.16 ^Aa^	6.86 ^ab^	0.343	0.008	0.140	0.780	0.047	0.198
	T2	5.78	5.89 ^B^	5.74						
*Ruminococcus*	T1	3.55	4.04	3.70 ^B^	0.568	0.037	0.672	0.847	0.393	0.609
	T2	5.53	5.46	7.68 ^A^						
*Christensenellaceae_R-7_group*	T1	4.53 ^Bb^	7.62 ^Aa^	6.32 ^ab^	0.331	0.246	0.542	0.032	0.176	0.020
	T2	6.28 ^A^	4.86 ^B^	5.16						
*UCG-004*	T1	3.36	1.85	3.01	0.183	0.181	0.177	0.116	0.791	0.215
	T2	2.42	2.25	2.08						
*Papillibacter*	T1	3.01	2.89 ^A^	2.39	0.203	0.003	0.823	0.634	0.794	0.252
	T2	1.56	1.16 ^B^	2.07						
*NK4A214_group*	T1	1.46	2.05	1.44	0.198	0.327	0.818	0.890	0.504	0.258
	T2	1.97	1.58	2.60						
*Saccharofermentans*	T1	1.51	2.25	3.10 ^A^	0.197	0.001	0.181	0.492	0.261	0.045
	T2	0.97	1.18	0.72 ^B^						
*UCG-005*	T1	1.07 ^B^	1.03 ^B^	1.23 ^B^	0.151	0.001	0.594	0.331	0.934	0.701
	T2	2.51 ^A^	2.01 ^A^	2.29 ^A^						
*Desulfovibrio*	T1	1.36 ^Aa^	0.57 ^Bb^	0.91 ^b^	0.188	0.286	0.051	0.429	0.035	0.001
	T2	0.48 ^Bb^	2.62 ^Aa^	0.65 ^b^						
*Berryella*	T1	1.35	1.77	1.21	0.069	0.409	0.099	0.093	0.243	0.413
	T2	1.56	1.63	1.48						
*Olsenella*	T1	2.60	1.38	1.23	0.168	0.113	0.215	0.982	0.176	0.083
	T2	1.05	1.60	1.01						

^1^ Groups HMF0, HMF7500, and HMF30000 indicate that the content of 5-HMF in the fermentation substrate is 0, 7500, and 30,000 mg/kg DM, respectively. ^2^ *p*, T: substrate; H: 5-HMF; L: linear; Q: quadratic; T × H: the interaction of substrate and 5-HMF. ^A–B^ In each column, mean values with different uppercase superscripts (A,B) indicate significant differences between dietary treatments (*p* < 0.05). ^a–b^ In each row, mean values with different lowercase superscripts (a–b) indicate significant differences among 5-HMF supplementation levels (*p* < 0.05).

## Data Availability

All raw data and sequencing information can be requested by contacting the corresponding author Liwen He (helw@cau.edu.cn).
